# Vorinostat Corrects Cognitive and Non-Cognitive Symptoms in a Mouse Model of Fragile X Syndrome

**DOI:** 10.1093/ijnp/pyab081

**Published:** 2021-11-17

**Authors:** Qi Ding, Xueting Wu, Xuan Li, Hongbing Wang

**Affiliations:** 1 Department of Physiology Michigan State University, East Lansing, Michigan, USA; 2 Neuroscience Program Michigan State University, East Lansing, Michigan, USA; 3 Key Laboratory of Synthetic Biology, CAS Center for Excellence in Molecular Plant Sciences, Institute of Plant Physiology and Ecology, Chinese Academy of Sciences, Shanghai, China

**Keywords:** Drug repurposing, fragile X syndrome, therapeutics, transcriptome analysis, vorinostat

## Abstract

**Background:**

Fragile X syndrome (FXS) is caused by mutations in the *FMR1* gene. It is a form of heritable intellectual disability and autism. Despite recent advance in elucidating disease mechanisms, there is no efficacious medication. Because de novo drug development is a lengthy process, repurposing the existing FDA-approved drugs offers an opportunity to advance clinical intervention for FXS. Our previous study with transcriptome analysis predicts potential therapeutic effects of vorinostat on FXS.

**Methods:**

We analyzed the vorinostat-induced transcriptome changes and confirmed its similarity to that induced by trifluoperazine, which was previously shown to correct pathological outcomes associated with FXS. To validate the therapeutic efficacy, we examined vorinostat’s effect on correcting the key behavioral and cellular symptoms in a mouse model of FXS.

**Results:**

We found that vorinostat restores object location memory and passive avoidance memory in the *Fmr1* knockout mice. For the non-cognitive behavioral symptoms, vorinostat corrected the autism-associated alterations, including repetitive behavior and social interaction deficits. In the open field test, vorinostat dampened hyperactivity in the center area of the arena. Surprisingly, vorinostat did not correct the abnormally elevated protein synthesis in cultured *Fmr1* knockout hippocampal neurons, suggesting that different aspects of pathological outcomes may respond differently to a specific therapeutic intervention.

**Conclusions:**

We used the drug-induced transcriptome signature to predict new application of existing drugs. Our data reveal the therapeutic effects of the FDA-approved drug vorinostat in a mouse model of FXS.

**Significance Statement**

Fragile X syndrome (FXS) is a main cause of heritable intellectual disability and autism. There is no effective treatment. We analyzed the drug-induced transcriptome signature database and identified an FDA-approved drug, vorinostat, as a potential treatment strategy. We further validated the therapeutic effects on an array of FXS-associated symptoms in a mouse model. This study reveals a novel approach to repurpose the existing drugs and also advocates vorinostat as a practical treatment.

## Introduction

Fragile X syndrome (FXS) is a genetic disorder caused by mutations in the *FMR1* (fragile X mental retardation 1) gene (Santoro et al., 2012). As the most frequent mutation, the increased number of the CGG trinucleotide repeat in the 5’ non-coding region hampers gene transcription and leads to a significant reduction or lack of fragile X mental retardation protein (FMRP) expression (Peprah 2012; Santoro et al., 2012). The main symptoms of FXS patients include cognitive disability, hyperactivity, and autism-related behavior (Santoro et al., 2012; Sethna et al., 2014). Although the disease mechanisms still remain elusive, studies with the preclinical animal models, which lack the expression of FMRP, have revealed potential therapeutic targets. It is recognized that FMRP interacts with its mRNA targets (Brown et al., 2001; Darnell et al., 2011) and predominantly suppresses translation of its mRNA targets (Darnell and Klann 2013). Another cellular abnormality is that lack of FMRP causes alteration of neuronal signaling, including the exaggerated metabotropic glutamate receptor 5, extracellular signal-regulated kinase ½, phosphoinositide 3-kinase, and S6 kinase 1 activity (Dolen et al., 2010; Osterweil et al., 2010; Sethna et al., 2014). Notably, genetic reduction of these signaling molecules corrects the elevation of global protein synthesis and certain behavioral symptoms in the FXS mouse model (i.e., *Fmr1* knockout [KO] mice) (Dolen et al., 2007; Bhattacharya et al., 2012; Gross et al., 2015). Further, candidate drugs that show inhibition activity against these signaling molecules exhibit therapeutic efficacy in *Fmr1* KO mice (Yan et al., 2005; Osterweil et al., 2013; Bhattacharya et al., 2016; Sethna et al., 2017; Gross et al., 2019; Ding et al., 2020a). Nevertheless, efficacious medication is not available yet.

Toward achieving clinical treatment for FXS, the development of brand-new drugs may easily take numerous years. Compared with de novo drug development, repurposing the existing FDA-approved drugs, of which the pharmacology and safety profile are available, is an attractive strategy. The main traditional approaches of drug repurposing usually depend on the outcome of high-throughput screening (Ashburn and Thor 2004) or knowledge of drug structure and mechanism of action and are sometimes driven by surprising observation of drug effects on new clinical symptoms. More recently, computational comparison of drug-induced transcriptome profiles has been proposed as a non-structure–based in-silico screening of similarity drugs (Lamb et al., 2006; Qu and Rajpal 2012; Iskar et al., 2013). It has been proposed that similarity of drug-induced transcriptome changes would suggest similarity of drug effects. By using computational analysis with transcriptome data in the Connectivity Map (CMap) database, our recent study predicts that the FDA-approved drug vorinostat may have therapeutic effects to correct FXS-associated symptoms (Ding et al., 2020b). However, to date, the transcriptome-based drug repurposing has not been applied for the treatment of neurological disorders, and the predicted efficacy of vorinostat requires empirical validation.

Vorinostat, also known as suberanilohydroxamic acid, is currently used to treat cancers, including cutaneous T-cell lymphoma and advanced non-small-cell lung carcinoma. Although as a small chemical compound, vorinostat should have promiscuous pharmacological activities, its inhibition activity against class I, II, and IV histone deacetylases (HDAC) is well recognized (Marks and Dokmanovic 2005). Consistently, 1 cellular outcome following vorinostat treatment is the increase of acetylation of various proteins, including histones. Vorinostat also shows neuroprotective effects in the central nervous system and is suggested to treat neurodegenerative disorders such as Alzheimer’s disease and Huntington disease (Hockly et al., 2003; Falkenberg and Johnstone 2014; Benito et al., 2015). Because histone acetylation–induced epigenetic changes are involved in activity-dependent plasticity and learning (Guan et al., 2009), a well-recognized outcome of vorinostat treatment is the enhancement of memory and cognition (Benito et al., 2015).

In this study, we examined the effects of vorinostat in the *Fmr1* KO mice. We found that vorinostat corrected deficits in object location memory and passive avoidance memory. Interestingly, it also corrected non-cognitive behavioral symptoms, including repetitive behavior, social interaction deficits, and a specific aspect of locomotion alteration. Our study provides evidence to support a new therapeutic action of vorinostat that is predicted by an unbiased transcriptome-based computational approach. It also advocates future tests with human clinical trials.

## Materials and Methods

### Comparison of Vorinostat-Induced Transcriptome Changes With Other Drugs in the CMap Database

The search for drugs/compounds that induce similar transcriptome changes to that of vorinostat in the CMap database was performed as described (Ding et al., 2020b). In brief, we first obtained microarray data sets of MCF7, PC3, and HL60 cells, which are the main cell lines included in the CMap database (http://www.broadintitute.org/cmap/), treated with vorinostat and the corresponding vehicle controls. The differential gene expression analysis was conducted, and the top 500 upregulated and downregulated genes were retained as the signature for further query (Irizarry et al., 2003; Gautier et al., 2004; Ding et al., 2020b). The transcriptome signature of vorinostat was uploaded to the CMap query page and used to search for compounds that induce similar or oppositional transcriptome changes in the corresponding cell line (i.e., MCF7, PC3, and HL60) (Lamb et al., 2006). The drug similarity ranking is based on permutation p, which is computed based on the Kolmogorov-Smirnov statistics (detailed procedure is described in its online help page: https://portals.broadinstitute.org/cmap/). The similarity compounds/drugs are listed with their *P* values in ascending order and are presented in supplementary Tables 1–3. Similarity drugs with a *P* > .05 in each cell line are not included.

### Animals

Young adult male mice at 2.5 to 3.5 months of age were used for behavioral examinations. Primary hippocampal neurons were obtained using postnatal day 0 mice. The *Fmr1* KO and their wild-type (WT) littermates are on the C57BL/6 background. The animals were maintained under a 12-hour-light/-dark cycle and had free access to food and water. The behavioral tests were performed between Zeitgeber time 4 and 8. Animals were used once for a particular examination and not repeatedly used for multiple tests. The Institutional Animal Care and Use Committee approved all procedures, which follow the international guidelines for the use and care of laboratory animal.

### Behavioral Examinations

For the examination of object location memory, mice were habituated to the training chamber without any object for 10 minutes on 3 consecutive days. Mice were then trained by a 10-minute exposure to the training chamber holding 2 objects at different locations (Fig. 2A), during which the mice freely explored the chamber and interacted with the objects. Twenty-four hours after training, the trained mice were tested by a 10-minute re-exposure to the same chamber with 1 object at the same location and 1 object at a new location (Fig. 2A). During training and testing, time spent in interacting with each object was recorded. The percent of preference was determined by the time spent with each object divided by the total time spent with both objects. Object interaction was manually scored.

For the examination of passive avoidance memory, mice were trained as described in our previous studies (Ding et al., 2014). During training, mice received a mild foot shock (0.7 mA for 2 seconds) immediately after entering the dark chamber. Then, the trap door connecting the light and dark chamber was closed. Mice stayed in the dark chamber for 30 seconds without being able to go back to the light chamber and were then returned to their home cage. Twenty-four hours later, the trained mice were reintroduced to the training chamber; crossover latency (i.e., the time elapsed until the mice crossed over and entered the dark chamber) was recorded. When there was no crossover beyond 600 seconds, the examination was terminated, and a crossover latency of 600 seconds was used for those specific mice. Crossover latency was manually scored with an electronic timer.

For the examination of activity in the light/dark box (Ding et al., 2014; Sethna et al., 2017), mice were introduced to a chamber that consisted of the connected light and dark compartments. Mice were first put in the dark compartment. Two minutes later, the trap door connecting the light and dark compartments was opened. During the 5-minute examination, the number of transitions between the 2 compartments and the time spent in the light compartment were recorded. The parameters involved in the light/dark box activity were scored manually.

For the examination of activity in the open field chamber (Coulbourn Instruments, Holliston, MA, USA), mice were allowed to freely explore the chamber for 60 minutes. The locomotor activity, as measured by ambulatory travel distance, in the whole chamber as well as in the center area was recorded and automatically scored by the TruScan software (Coulbourn Instruments).

For the examination of social interaction, mice were subjected to the 3-chamber social interaction test as described in our previous studies (Sethna et al., 2017; Ding et al., 2020b). Mice were first allowed to freely explore the testing box, which consisted of 3 connected chambers of the same size with a 5-cm opening in the partition wall. Five minutes later, a novel stimulus mouse in a wire enclosure and an empty wire enclosure were placed in the “social” and “non-social” chamber, respectively. During the 10-minute examination, the total time spent in the social and non-social chambers and the time spent in sniffing the stimulus mouse enclosure were recorded. The parameters involved in the 3-chamber social interaction test were scored manually.

### In Vivo Administration of Vorinostat

Vorinostat (Sigma-Aldrich, St. Louis, MO, USA) was prepared in the vehicle (10% dimethyl Sulfoxide [DMSO]) and i.p. injected into mice. Based on the established doses, at which previous in vivo studies show that such treatments are sufficient to improve cognitive functions in mice (Guan et al., 2009; Benito et al., 2015), vorinostat was administered at 50 mg/kg (Basu et al., 2019). The drug effects were first examined with a single i.p. injection. For certain behavioral symptoms not responsive to a single vorinostat administration, the effect of extended treatment (daily injection at 50 mg/kg for 2 weeks) was examined. Thirty minutes after the single injection or the last daily injection, mice were subjected to object location and passive avoidance training or examined for behavioral activity in the open field, light/dark box, and social interaction (see administration regime in Figs. 2A, 2D1, 2E1, 3A1, 3B1, 4A1, 4B1). The control groups were treated with vehicle injection.

### Examination of Protein Synthesis in Neurons

Primary hippocampal neurons were obtained from postnatal day 0 WT and *Fmr1* KO mice and maintained in vitro. To determine protein synthesis with the SUnSET method (Schmidt et al., 2009), 14 days in vitro neurons were incubated with 5 μg/mL puromycin (Sigma, St. Louis, MO, USA; Cat #P8833) for 30 minutes and then harvested in Buffer H (50 mM beta-glycerophosphate, 1.5 mM EGTA [ethylene glycol-bis(β-aminoethyl ether)-*N*,*N*,*N*′,*N*′-tetraacetic acid], 0.1 mM Na_3_VO_4_, 1 mM DTT [dithiothreitol]). After the determination of total protein concentration, the samples were examined by western blot with anti-puromycin antibody (KeraFAST, Boston, MA, USA; Cat # EQ0001; 1:1000). The effects of vorinostat on protein translation were determined with neurons that were first treated with vorinostat (20 and 40 μM) for 30 minutes followed by a 30-minute puromycin incubation. The relative amount of loading was determined by β-actin. The intensity of the immuno-signal was analyzed by the ImageJ software (NIH, MD, USA).

### Examination of Histone Acetylation

Samples were collected from 14 days in vitro hippocampal neurons, hippocampus of 3-month-old mice, and prefrontal cortex of 3-month-old mice. The level of histone acetylation was examined by western blot with antibodies against total and acetylated histone proteins H2B and H3 (Cell Signaling, Danvers, MA, USA; 1:1000). The relative amount of loading was determined by β-actin. The intensity of the immuno-signal was analyzed by the ImageJ software (NIH).

### Data Collection and Statistics

Mice were randomly assigned to vehicle and drug treatment groups, which were not disclosed before data analysis. Mice from multiple litters were used to avoid pseudo replication. The detailed information of the number of litters is listed in supplementary Table 4. Data from all samples were included for analysis. All data are expressed as mean ± SEM along with each individual data point plotted in the figures. Data with normal distribution were analyzed by 2-sided Student’s *t* test or ANOVA. The crossover latency data for passive avoidance testing did not show normal distribution and were analyzed by Fisher’s exact test.

## Results

### In-Silico Screening of Vorinostat Similarity Drugs in the CMap Database

The transcriptome landscape reflects a particular aspect of molecular outcome in physiological and pathological conditions (Tasic et al., 2016; So et al., 2017; Gandal et al., 2018). We recently found that transcriptome changes in the *Fmr1* KO neurons can successfully predict therapeutic interventions. Among the predicted drugs, an FDA-approved antipsychotics trifluoperazine causes transcriptome alteration oppositional to that caused by FMRP deficiency. It was further demonstrated that trifluoperazine corrects the key FXS-associated symptoms in the *Fmr1* KO mice (Ding et al., 2020b). Moreover, computational analysis of the trifluoperazine-induced transcriptome signature revealed other similarity drugs, predicting that those similarity drugs may be repurposed to treat FXS. In addition to trifluoperazine, 3 other antipsychotics are among the top 10 similarity drugs. Interestingly, vorinostat, which is a known HDAC inhibitor, is the third-ranked similarity drug following the 2 antipsychotics (Ding et al., 2020b).

**Figure 1.**
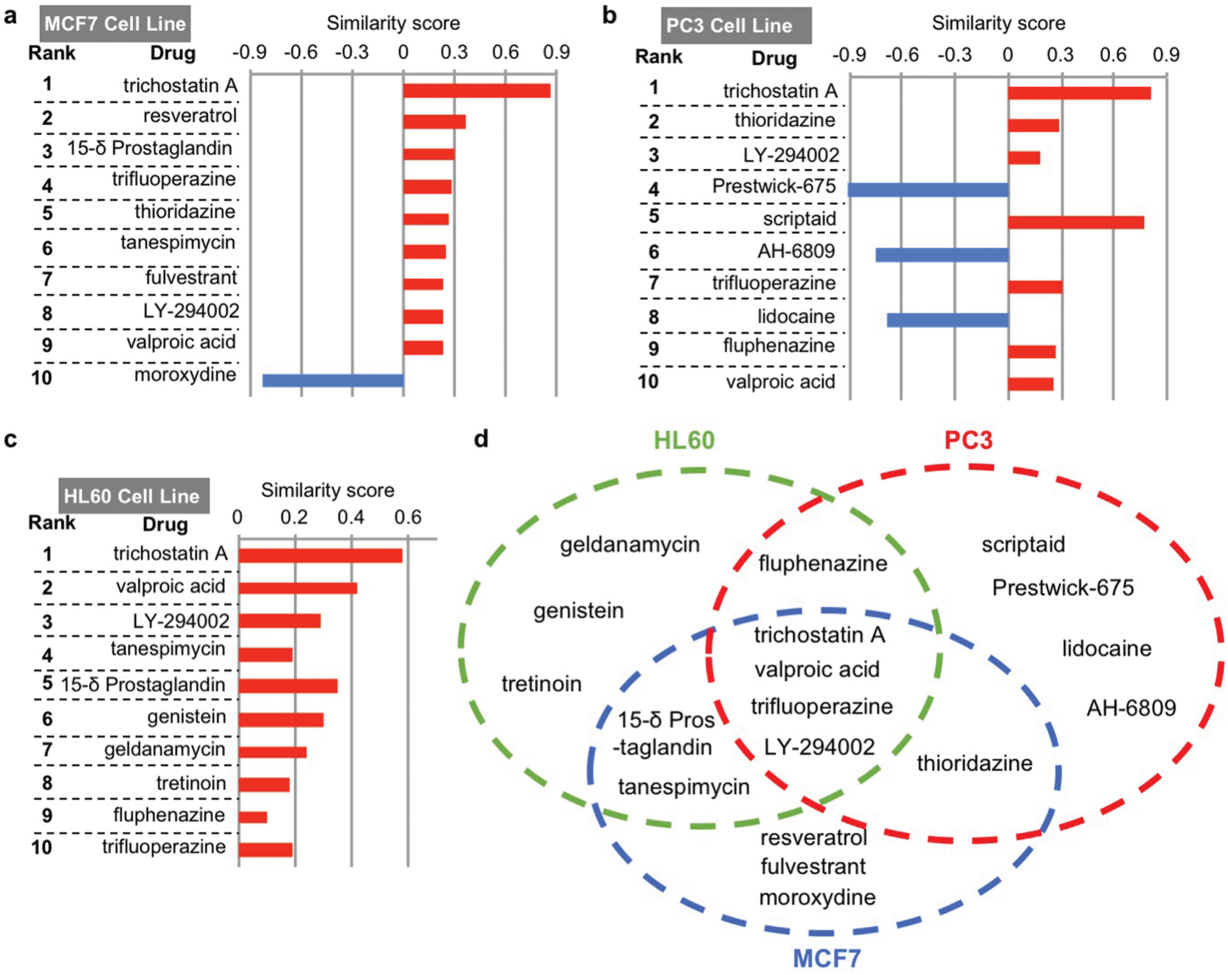
Similarity drugs of vorinostat identified by the drug-induced transcriptome changes. The top 10 similarity drugs/compounds of vorinostat, along with the similarity scores, in MCF7, PC3, and HL60 cell lines, are shown in A, B, and C, respectively. Some similarity drugs/compounds of vorinostat are identified from a unique cell line, as indicated in D. Some similarity drugs/compounds induce similar transcriptome changes to that of vorinostat in multiple cell lines (D).

It is important to examine whether the transcriptome-based analysis can also identify trifluoperazine as a similarity drug of vorinostat. Here, we further used vorinostat-induced transcriptome perturbations as a query to identify similarity drugs in the CMap database (Broad Build 02 database, http://www.broadinstitute.org), which contains over 7000 reference gene signatures altered by 1309 compounds/perturbagens. Because the gene signatures were characterized in 3 major cell lines (i.e., MCF7, PC3, and HL60) in the database, we performed a computational analysis to search for similarity drugs within each cell line. The transcriptome signature of vorinostat in each cell line identified drugs showing significant positive and negative similarity scores (i.e., similarity mean with a *P* < .05) (supplementary Tables 1 –3). Notably, there is an overlap of similarity drugs among the 3 cell lines, indicating a certain degree of conservation of transcriptional responses to vorinostat. Among the top 10 ranked compounds (Fig. 1A–C), 4 drugs are the common vorinostat similarity drugs identified from all 3 cell lines (Fig. 1D). These 4 similarity drugs include 2 HDAC inhibitors (i.e., trichostatin A and valproic acid), trifluoperazine, and a known phosphatidylinositol 3-kinase inhibitor, LY-294002. These data further support that, based on their effects on transcriptome signature, vorinostat and trifluoperazine are mutual similarity drugs and may have similar action to correct FXS-associated symptoms. To validate the in silico prediction of drug action, we examined the effects of vorinostat in a mouse model of FXS.

### Vorinostat Corrects Cognitive Deficits in the *Fmr1* KO Mice

**Figure 2.**
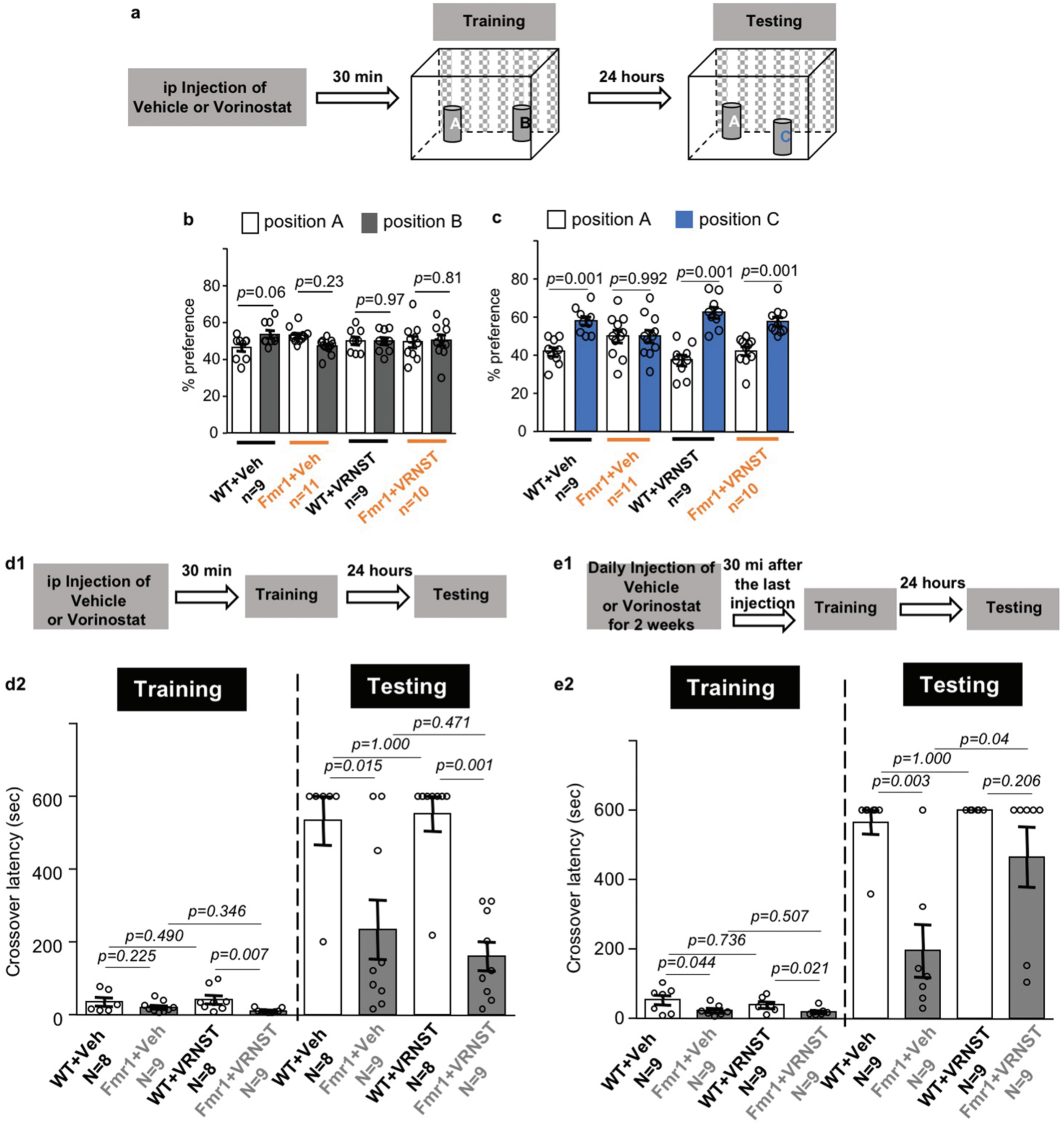
Effects of vorinostat on the correction of object location memory and passive avoidance memory in the *Fmr1* KO mice (Fmr1). (A) Drug administration and object location memory paradigm. Following a single i.p. injection with vehicle (Veh) or vorinostat (VRNST), wild-type (WT) and Fmr1 mice were subject to a training chamber with certain spatial cues and allowed to explore the 2 objects at location A and B. During testing, the trained mice were reintroduced to the training chamber and allowed to interact the 2 objects at location A and C. (B) Mouse preference to the object at locations A and B during training. (C) Mouse preference to the object at locations A and C during testing. (D1, E1) Timeline of drug administration, training, and testing for passive avoidance memory. WT and Fmr1 mice were first injected with Veh or VRNST and then received passive avoidance training. The trained mice were tested 24 hours later. (D) Mice were trained after a single injection of Veh or VRNST. (E) Mice were first injected with Veh or VRNST daily for 2 weeks and then trained 30 minutes after the last injection. Crossover latency during training and testing was recorded (D2 and E2). Data are presented as mean ± SEM. The *P* values in B and C were determined by 3-way ANOVA followed by post hoc pairwise comparison with Holm-Sidak adjustment. The *P* values for passive avoidance training data (the left panel in D2 and E2) were determined by 2-way ANOVA followed by post hoc pairwise comparison with Holm-Sidak adjustment. The *P* values for the passive avoidance testing data (the right panel in D2 and E2) were determined by the Fisher exact test.

Recapitulating the intellectual deficits in human FXS patients, the *Fmr1* KO mice show compromised cognitive function (Ding et al., 2014; Sethna et al., 2014). We first examined the effects of vorinostat on object location memory (Fig. 2A). We administered vorinostat at 50 mg/kg, which was established in previous studies and effective to alter in vivo brain function in mice (Basu et al., 2019). Following an acute single-drug treatment, the vehicle- and vorinostat-treated WT and *Fmr1* KO mice showed comparable preference to objects at locations A and B (Fig. 2B) (object location effect: *F*_1,70_ = 0.133, *P* = .716) during the training session. During testing, the vehicle-treated WT, but not *Fmr1* KO mice, showed preference for the object at the novel location C (Fig. 2C) (object location effect: *F*_1,70_ = 52.032, *P* = .001), indicating that object location memory is compromised in *Fmr1* KO mice. In contrast, both the vorinostat-treated WT and *Fmr1* KO mice showed preference for the object at location C (Fig. 2C), indicating significant location memory formation.

We next examined the effects of vorinostat on passive avoidance memory (Fig. 2D1 and E1). Following an acute single administration of vorinostat, we found a genotype effect but no drug effect on crossover latency (genotype effect: *F*_1,30_ = 8.514, *P* = .007; drug effect: *F*_1,30_ = 0.022, *P* = .884; genotype × drug interaction: *F*_1,30_ = 1.358, *P* = .253; Fig. 2D2) during training. The vehicle-treated WT and *Fmr1* KO mice showed similar crossover latency, and the vorinostat-treated *Fmr1* KO mice showed less crossover latency than WT mice during training (Fig. 2D2). Still, there was no significant difference between vehicle- and vorinostat-treated *Fmr1* KO mice (Fig. 2D2). When tested 24 hours later, the vehicle-treated *Fmr1* KO mice displayed longer crossover latency than that during training. However, they showed less crossover latency than the vehicle-treated WT mice (Fig. 2D2), indicating impaired passive avoidance memory. For the vorinostat-treated groups, the passive avoidance memory was still impaired in *Fmr1* KO mice (Fig. 2D2). Thus, an acute and single administration of vorinostat failed to improve memory in the *Fmr1* KO mice.

Because a single vorinostat administration failed to correct passive avoidance memory, we further examined the effect of an extended treatment. We treated mice with repeated vorinostat administration, prior to training, by daily i.p. injection for 2 weeks (Fig. 2E1). There was a genotype effect but no drug effect on behavior during training (genotype effect: *F*_1,32_ = 10.201, *P* = .003; drug effect: *F*_1,32_ = 0.511, *P* = .480; genotype × drug interaction: *F*_1,32_ =  0.054, *P* = .817; Fig. 2E2). Repeated vorinostat restored passive avoidance memory in the *Fmr1* KO mice to the WT level, as indicated by the improved crossover latency during testing (Fig. 2E2).

### Therapeutic Effects of Vorinostat on Repetitive Behavior and Hyperactivity in the *Fmr1* KO Mice

**Figure 3.**
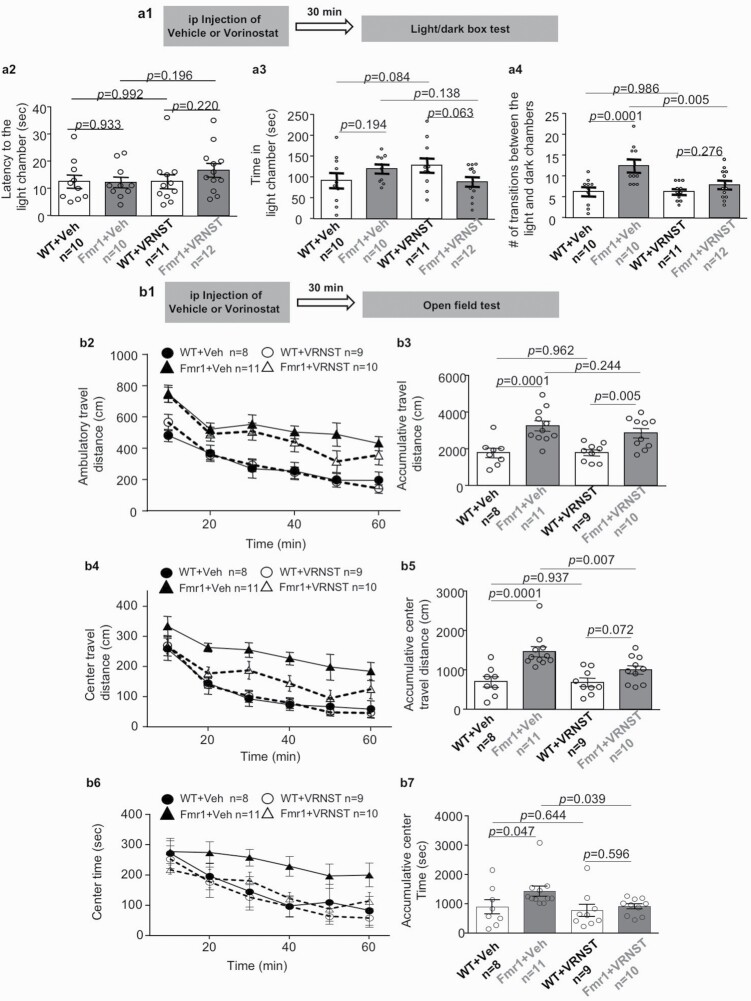
Effects of vorinostat on repetitive behavior and hyper-locomotion in the *Fmr1* KO (Fmr1) mice. Following a single administration with vehicle (Veh) or vorinostat (VRNST) (A1 and B1), wild-type (WT) and Fmr1 mice were subject to the light/dark box test (A1–4) and the open field test (B1–7). (A) During the light/dark box test, the mice were allowed to make transitional moves between the light and the dark chamber. The latency to exit the dark chamber (A2), time spent in the light chamber (A3), and number of transitional moves between the light and dark chambers (A4) are presented as mean ± SEM. (B) During the 60-minute open field test, ambulatory travel distance within the whole arena (B2 and B3), ambulatory travel distance in the center area (B4 and B5), and time spent in the center area (B6 and B7) were recorded. Activities for each 10-minute bin (B2, B4, and B6) and accumulative activity during the whole 60-minute testing (B3, B5, and B7) are presented as mean ± SEM. The *P* values were determined by 2-way ANOVA followed by post hoc pairwise comparison with Holm-Sidak adjustment.

We determined the effect of vorinostat on behavior in the light/dark box test (Fig. 3A1), during which the mice made repetitive transitional movements between the light and dark chambers. We found that the vehicle-treated WT and *Fmr1* KO mice show comparable latency to exit the dark chamber and enter the light chamber (Fig. 3A2) (genotype effect: *F*_1,39_ = 0.622, *P* = .435). They also showed similar preference and spent comparable time in the light chamber (Fig. 3A3) (genotype effect: *F*_1,39_ = 0.156, *P* = .695), indicating comparable anxiety-related activity in the light/dark test. Interestingly, the vehicle-treated *Fmr1* KO mice showed a higher number of repetitive transitions between the light and dark chambers (Fig. 3A4) (genotype effect: *F*_1,39_ = 12.932, *P* = .001). Administration of vorinostat normalized this hyperactive and repetitive transition behavior in the *Fmr1* KO mice (drug effect: *F*_1,39_ = 4.469, *P* = .041; genotype × drug interaction: *F*_1,39_ = 4.364, *P* = .043; Fig. 3A4) but had no effect on the latency (to enter the light chamber) (drug effect: *F*_1,39_ = 0.861, *P* = .359; Fig. 3A2) and the time spend in the light chamber (drug effect: *F*_1,39_ = 0.042, *P* = .840; Fig. 3A3).

We next determined the effect of vorinostat on locomotion behavior in the open field test (Fig. 3B1). The vehicle-treated *Fmr1* KO mice showed more locomotion activity than the vehicle-treated WT mice in the whole arena (genotype effect: *F*_1,34_ = 25.844, *P* = .001; drug effect: *F*_1,34_ = 0.573, *P* = .454; genotype × drug interaction: *F*_1,34_ = .685, *P* = .414; Fig. 3B2 and 3). They also showed higher locomotion activity (genotype effect: *F*_1,34_ = 19.996, *P* = .001; drug effect: *F*_1,34_ = 3.910, *P* = .056; genotype × drug interaction: *F*_1,34_ = 3.457, *P* = .072; Fig. 3B4 and B5) in the center area. Following vorinostat administration, the *Fmr1* KO mice did not show changes of locomotion in the whole arena (Fig. 3B2 and B3); they showed a reduction of locomotion in the center area (Fig. 3B4 and B5). Although the overall 2-way ANOVA analysis revealed no difference of occupancy time in the center area among the vehicle- and vorinostat-treated WT and *Fmr1* KO mice (genotype effect: *F*_1,34_ = 3.404, *P* = 0.074; drug effect: *F*_1,34_ = 3.190, *P* = .083; genotype × drug interaction: *F*_1,34_ = 1.195, *P* = .282; Fig. 3B6 and B7), pairwise comparison revealed that *Fmr1* KO mice spent more time in the center area, and the phenotype was corrected by vorinostat (Fig. 3B6 and B7).

### Therapeutic Effects of Vorinostat on Social Deficits in the *Fmr1* KO Mice

**Figure 4.**
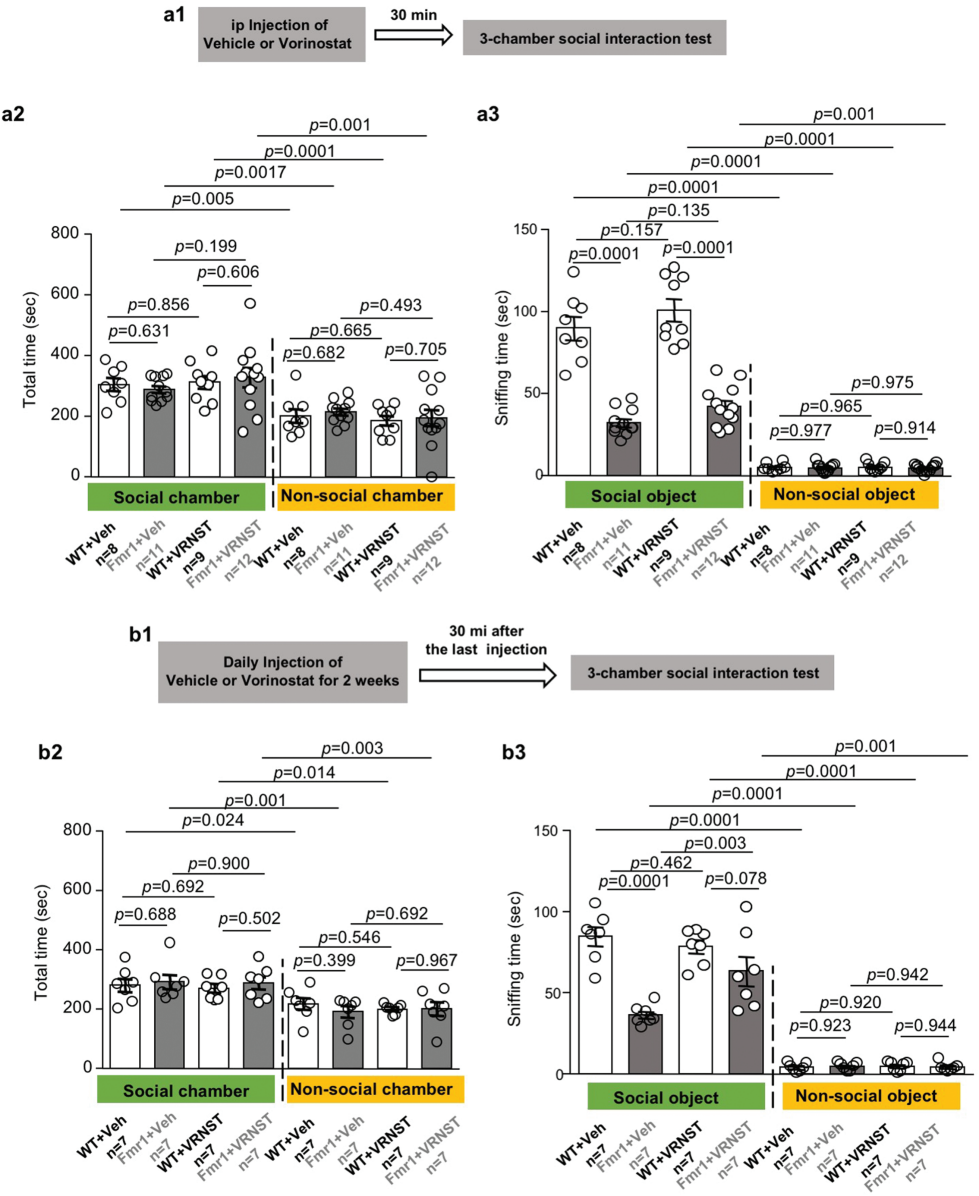
Effects of vorinostat on social interaction deficits in the *Fmr1* KO (Fmr1) mice. Following a single (A) or repeated (daily for 2 weeks) (B) administration with vehicle (Veh) or vorinostat (VRNST), wild-type (WT) and Fmr1 mice were subjected to the 3-chamber social interaction test (A1 and B1). During the 10-minute social interaction test, the total time spent in the social and non-social chamber (A2 and B2) as well as the time spent in direct interaction with the social (i.e., a stranger mouse in a wire enclosure) and the non-social object (i.e., a novel object in a wire enclosure) (A3 and B3) were recorded. Data are presented as mean ± SEM. The *P* values were determined by 3-way ANOVA followed by post hoc pairwise comparison with Holm-Sidak adjustment.

Following an acute and single vorinostat administration, we examined social interaction (Fig. 4A1). All groups of mice showed preference for the social vs the non-social chamber (chamber effect: *F*_1,72_ = 45.769, *P* = .001; Fig. 4A2). All groups spent similar time in the social chamber (genotype effect: *F*_1,36_ = 0.000, *P* = .995; Fig. 4A2) as well as in the non-social chamber (genotype effect: *F*_1,36_ = 0.262, *P* = .612; Fig. 4A2). Although all groups spent more time interacting with the social object (i.e., a stranger mouse in a wire enclosure) than the non-social object (i.e., a novel object in a wire enclosure) (object effect: *F*_1,72_ = 599.123, *P* = .0001; Fig. 4A3), the vehicle-treated *Fmr1* KO mice showed less interaction with the social object than the vehicle-treated WT mice (genotype effect: *F*_1,36_ = 137.711, *P* = .001; Fig. 4A3), indicating defective social interaction. A single vorinostat administration failed to rescue the social deficits in *Fmr1* KO mice (Fig. 4A3; genotype effect: *F*_1,36_ = 137.711, *P* < .001; drug effect: *F*_1,36_ = 4.386, *P* = .043; genotype × drug interaction: *F*_1,36_ =  0.01, *P* = .921).

Because a single vorinostat administration failed to correct social behavior deficits, we further examined the effect of an extended treatment. We next examined the effect of repeated vorinostat treatment on social interaction (Fig. 4B1). After daily injection with vorinostat for 2 weeks, we found that the vorinostat-treated *Fmr1* KO mice showed improvement in social interaction. The vorinostat-treated *Fmr1* KO mice and WT mice spent comparable time interacting with the social object (genotype effect: *F*_1,24_ = 29.767, *P* = .001; drug effect: *F*_1,24_ = 3.219, *P* = .085; genotype × drug interaction: *F*_1,24_ = 8.127, *P* = .009; Fig. 4B3). The interaction with the non-social object was not affected by the repeated vorinostat treatment (Fig. 4B3; genotype effect: *F*_1,24_ = 0.05, *P* = .942; drug effect: *F*_1,24_ = 0.05, *P* = .942; genotype × drug interaction: *F*_1,24_ = 0.261, *P* = .614).

### Effects of Vorinostat on Protein Synthesis

**Figure 5.**
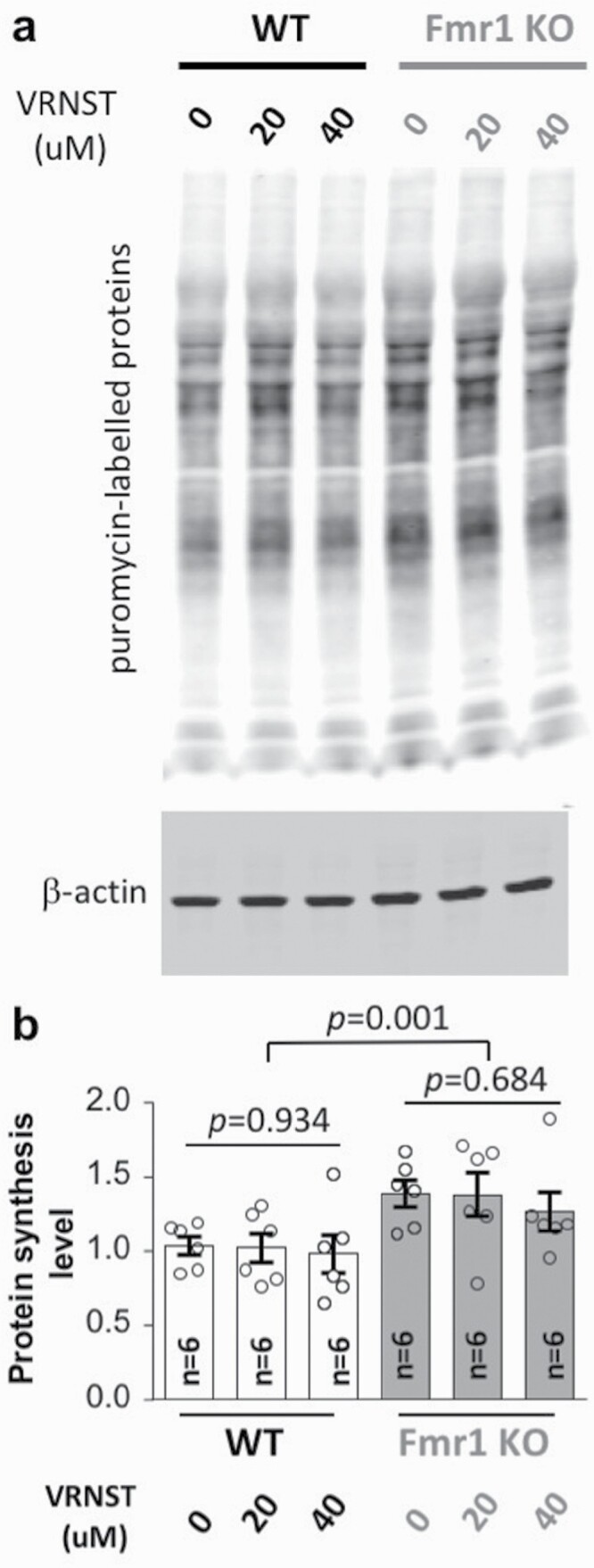
Vorinostat does not correct the elevated protein synthesis in the *Fmr1* KO neurons. Wild-type (WT) and *Fmr1* KO hippocampal neurons were treated with vehicle or vorinostat (VRNST) at 20 and 40 μM, as indicated, for 30 minutes, following which puromycin was applied for 30 minutes. Samples were then collected and subjected to western-blot analysis. Representative images are presented in A. Quantification is shown as mean ± SEM in B (for all groups, n = 6: triplicates from 2 independent primary neuronal cultures). The *P* values were determined by 1-way and 2-way ANOVA followed by post hoc pairwise comparison with Holm-Sidak adjustment.

The abnormally elevated protein synthesis has been recognized as a prominent aspect of cellular pathology associated with FXS (Santoro et al., 2012; Sethna et al., 2014). We confirmed that the *Fmr1* KO hippocampal neurons display a higher level of new protein synthesis than WT hippocampal neurons (genotype effect: *F*_1,30_ = 13.060, *P* = .001; drug effect: *F*_2,30_ = 0.386, *P* = .683; genotype × drug interaction: *F*_2,30_ = 0.067, *P* = .935; Fig. 5). However, vorinostat failed to suppress protein synthesis in both *Fmr1* KO (*F*_2,30_ = 0.384, *P* = .684) and WT hippocampal neurons (*F*_2,30_ = 0.068, *P* = .934; Fig. 5).

### Correction of Behavioral Symptoms Is Not Directly Mediated by Pharmacological Inhibition of HDAC

**Figure 6.**
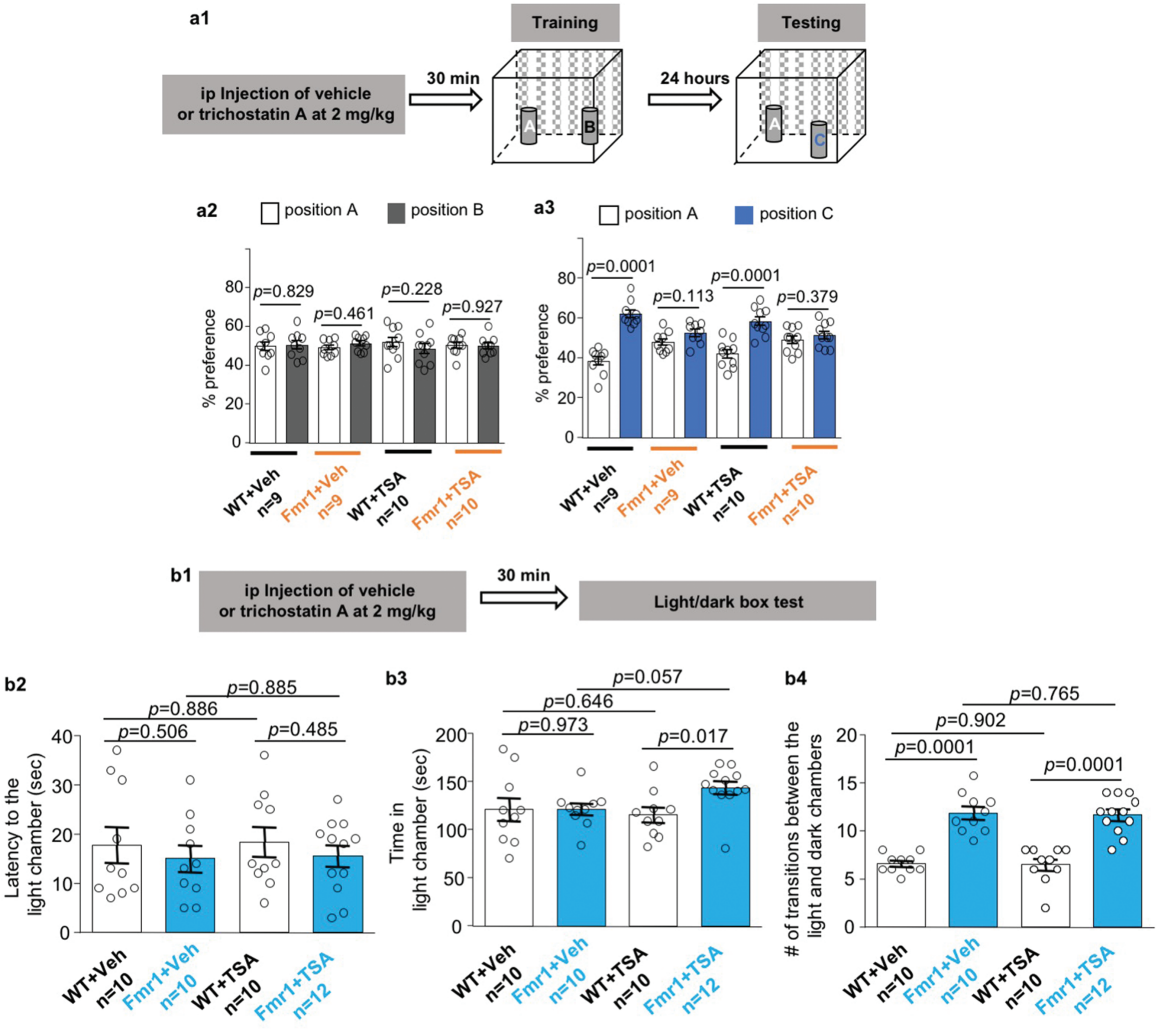
Trichostatin A does not affect object location memory and light/dark box behavior in the *Fmr1* KO (Fmr1) mice. Wild type (WT) and Fmr1 mice were injected with vehicle (Veh) or trichostatin A at 2 mg/kg (TSA) (A1 and B1). Thirty minutes later, mice were trained to learn object location (A) or subjected to a light/dark box test (B). (A2) Mouse preference to the object at locations A and B during training. (A3) Mouse preference to the object at locations A and C during testing. (B) During the light/dark box test, the mice were allowed to make transitional moves between the light and the dark chamber. The latency to exit the dark chamber (B2), time spent in the light chamber (B3), and number of transitional moves between the light and dark chambers (B4) were recorded. Data are presented as mean ± SEM. The *P* values were determined by 3-way (A) or 2-way ANOVA (B) followed by post hoc pairwise comparison with Holm-Sidak adjustment.

Because the main known pharmacological action of vorinostat is HDAC inhibition, we wondered whether the observed therapeutic efficacy is due to pharmacological inhibition of HDAC. To test this possibility, we examined the therapeutic effect of another well-known HDAC inhibitor trichostatin A (Bieliauskas and Pflum 2008). We chose to examine the effect of trichostatin A on object location memory and light-dark box behavior because the abnormalities of these behaviors are more sensitive to vorinostat treatment and can be corrected by a single administration of vorinostat. Trichostatin A at 2 mg/kg, which is an effective dose to improve cognitive functions in a mouse model of Rubinstein-Taybi syndrome (Korzus et al., 2004), failed to improve object location memory in *Fmr1* KO mice (Fig. 6A). Trichostatin A at 2 mg/kg was also not effective to dampen the repetitive transition behavior in *Fmr1* KO mice in the light/dark test (Fig. 6B). Interestingly, trichostatin A increased the light chamber occupancy time, which is not a genotype-specific alteration, in *Fmr1* KO mice (Fig. 6B3).

Previous studies have also used higher doses to examine the in vivo effect of trichostatin A. We wondered whether trichostatin A at a higher dose could exert therapeutic efficacy. We found that increasing the dose of trichostatin to 10 mg/kg was also not effective to improve object location memory (supplementary Fig. 1), dampen repetitive transition in the light/dark test (supplementary Fig. 2), and normalize locomotor hyperactivity (supplementary Fig. 3) in *Fmr1* KO mice.

**Figure 7.**
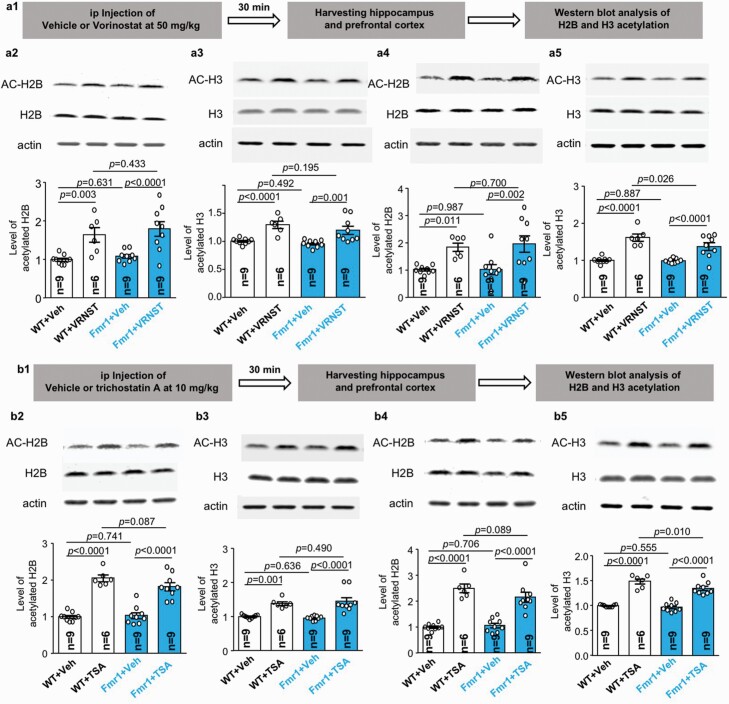
Effects of vorinostat and trichostatin A on histone acetylation in mouse brain. Wild-type (WT) and *Fmr1* KO mice were injected with vorinostat (50 mg/kg) (A) or trichostatin A (10 mg/kg) (B) or vehicle (A and B). Thirty minutes after the injection, hippocampus (A2, A3, B2, B3) and prefrontal cortex (A4, A5, B4, B5) were harvested. Western blot was used to determine the level of acetylated H2B (A2, A4, B2, B4) and acetylated H3 (A3, A5, B3, B5), which were normalized to the total level of the respective histone proteins. Data are presented as mean ± SEM. The *P* values were determined by 2-way ANOVA (A2: genotype effect: *F*_1,29_ = 0.838, *P* = .36; drug effect: *F*_1,29_ = 26.320, *P* < .001; genotype × drug interaction: *F*_1,29_ = 0.072, *P* = .790) (A3: genotype effect: *F*_1,29_ = 2.109, *P* = .157; drug effect: *F*_1,29_ = 30.665, *P* < .001; genotype × drug interaction: *F*_1,29_ = 0.275, *P* = .604) (A4: genotype effect: *F*_1,29_ = 0.090, *P* = .766; drug effect: *F*_1,29_ = 18.509, *P* < .001; genotype × drug interaction: *F*_1,29_ = 0.078, *P* = .783) (A5: genotype effect: *F*_1,29_ = 3.418, *P* = .075; drug effect: *F*_1,29_ = 59.934, *P* < .001; genotype × drug interaction: *F*_1,29_ = 2.746, *P* = .108) (B2: genotype effect: *F*_1,29_ = 1.205, *P* = .281; drug effect: *F*_1,29_ = 114.461, *P* < .001; genotype × drug interaction: *F*_1,29_ = 2.380, *P* = .134) (B3: genotype effect: *F*_1,29_ = 0.041, *P* = .841; drug effect: *F*_1,29_ = 43.080, *P* < .001; genotype × drug interaction: *F*_1,29_ = 0.706, *P* = .408) (B4: genotype effect: *F*_1,29_ = 1.117, *P* = .299; drug effect: *F*_1,29_ = 98.437, *P* < .001; genotype × drug interaction: *F*_1,29_ = 2.446, *P* = .129) (B5: genotype effect: *F*_1,29_ = 5.957, *P* = .021; drug effect: *F*_1,29_ = 158.015, *P* < .001; genotype × drug interaction: *F*_1,29_ = 2.702, *P* = .111) followed by post hoc pairwise comparison with Holm-Sidak adjustment.

We further found that levels of the acetylated histone proteins H2B (Fig. 7A2 and A4, B2 and B4) and H3 (Fig. 7A3 and A5, B3 and B5) are normal in the hippocampus (Fig. 7A2 and A3, B2 and B3) and prefrontal cortex (Fig. 7A4 and A5, B4 and B5) of *Fmr1* KO mice as well as in primary *Fmr1* KO hippocampal neurons (supplementary Fig. 4). Notably, Li et al. found that FMRP deficiency causes reduction of HDAC1 (Li et al., 2018), implicating that a particular aspect of HDAC activity may be decreased in FXS. Further, although only vorinostat, but not trichostatin A, corrected the FXS-associated behavioral symptoms, they both effectively caused significant increase of H2B (Fig. 7A2 and A4, B2 and B4) and H3 acetylation (Fig. 7A3 and A5, B3 and B5) in the hippocampus (Fig. 7A2 and A3, B2 and B3) and prefrontal cortex (Fig. 7A4 and A5, A4 and B5) of WT and *Fmr1* KO mice. Our data and previous research suggest that the therapeutic effect of vorinostat is unlikely due to its inhibition activity against HDAC.

## Discussion

Although there are no efficacious therapeutics, de novo drug development for FXS treatment is still in its infancy and encountered significant obstacles (Berry-Kravis et al., 2018). One alternative and efficient approach is to repurpose the existing FDA-approved drugs (Tranfaglia et al., 2019). In this study, we used the CMap drug-induced transcriptome database to predict that vorinostat and trifluoperazine, which has been recently found to correct FXS-associated symptoms in a mouse model (Ding et al., 2020b), are mutually similar drugs. The therapeutic efficacy of vorinostat in correcting a variety of behavioral symptoms is validated with the *Fmr1* KO mice.

The use of a holistic analysis of transcriptome signature to predict therapeutic strategy for neurological disorders has been recently proposed but not empirically examined (So et al., 2017). One application is to compare the disease-associated transcriptome signature with drug-induced transcriptome signatures. The value of this application is implicated by the fact that transcriptome signature associated with bipolar disorder and schizophrenia can predict drugs, and some of the predicted drugs are already in clinical use to treat these disorders (So et al., 2017; Gandal et al., 2018). As the first empirical attempt, we screened the CMap database with the FXS-associated transcriptome signature and identified trifluoperazine as a therapeutic reagent to treat symptoms in the *Fmr1* KO mice (Ding et al., 2020b). Another important application of the transcriptome-based therapeutic prediction is to compare the transcriptome signatures associated with different drugs and chemical compounds (Lamb et al., 2006; Qu and Rajpal 2012). Using the trifluoperazine-induced transcriptome change as query identified vorinostat as a top-ranked similarity drug of trifluoperazine (Ding et al., 2020b). In this study, using the vorinostat-induced transcriptome change as a query also identified trifluoperazine as a top-ranked similarity drug of vorinostat. The transcriptome similarity predicts that vorinostat may have similar therapeutic effects to that of trifluoperazine and be useful to treat specific symptoms associated with FXS. Empirically, this study validates the transcriptome-based approach to identify new drug action and repurpose vorinostat.

It is important to note that the vorinostat- and trifluoperazine-induced transcriptome changes share similarities but are not identical. Depending on the cell types, scores (i.e., similarity mean) underlying the similarity between vorinostat and trifluoperazine are 0.282, 0.307, and 0.19 (Fig. 1; supplementary Tables 1–3). Because a score of 1 reflects being identical, and a score of −1 reflects being oppositional, it is anticipated that vorinostat and trifluoperazine should have both common and different pharmacological actions. As far as the correction of behavior symptoms is concerned, vorinostat and trifluoperazine have similar but not identical effects. For example, while a single administration of trifluoperazine rescues passive avoidance memory and social deficits (Ding et al., 2020B), correction of these deficits requires repeated dosing of vorinostat (Fig. 2E and 4B). In contrast, vorinostat but not trifluoperazine corrected certain aspects of hyperactive locomotion in the open field (Fig. 3B4 and 3B5, and [Ding et al., 2020B]). Regarding that trifluoperazine is not sufficient to rescue all FXS symptoms, it is significant to identify similarity drugs (e.g., vorinostat) and their new therapeutic efficacy.

It has been recognized that HDAC inhibitors may be considered to improve learning and memory in animal models of cognition impairment (Guan et al., 2009; Benito et al., 2015; Sharma et al., 2015). The effect of HDAC inhibitors on non-cognitive functions such as repetitive behavior, hyperactivity, and social interaction has not been recognized and appreciated. Here, we found the therapeutic effects of vorinostat to correct both cognitive and non-cognitive symptoms in *Fmr1* KO mice. Interestingly, vorinostat only affected behavior in the *Fmr1* KO but not WT mice. Because the acetylation levels of H2B and H3 are normal in *Fmr1* KO neurons, it is not straightforward to attribute the therapeutic effect of vorinostat to its HDAC inhibition activity. Further, another HDAC inhibitor, trichostatin A, failed to rescue deficits of either cognitive or non-cognitive functions. We speculate that the therapeutic effect of vorinostat is not mediated through its pharmacological action against HDAC. Currently, we are not able to identify relevant and functional target of vorinostat; it remains unclear whether and how vorinostat impinges on the molecular abnormalities such as altered neuronal signaling in FXS. We acknowledge that vorinostat does not correct all symptoms associated with FXS. Different aspects of pathological outcomes respond differently to vorinostat and remain to be investigated with future studies. With regard to mechanism of action, this study does not elucidate direct and specific molecular targets of vorinostat.

Notably, although vorinostat corrects certain FXS-associated behavior symptoms, it does not normalize the elevated global protein synthesis in cultured *Fmr1* KO hippocampal neurons. This is intriguing and suggests that, at least to a certain degree, elevated protein synthesis may not be absolutely linked to all behavioral abnormalities. A recent study found that human FXS samples show various levels of protein synthesis, and fibroblasts derived from some FXS patients display normal translation (Jacquemont et al., 2018). However, we acknowledge the limitation of using cultured hippocampal neurons to determine protein translation. One complication is that it is not clear whether the elevated translation is universal or restricted to specific brain regions and cell types. Because the abnormally elevated protein synthesis in FXS is mostly reported in the hippocampus and the cognitive function examined in this study depends on the hippocampus, we examined the effect of vorinostat in hippocampal neurons. We acknowledge that protein synthesis in neurons collected from other brain regions (e.g., striatum and various cortical regions) may be regulated differently and affected by vorinostat. Alternatively, vorinostat may dampen the translation of particular FMRP target mRNAs rather than affecting overall protein synthesis. These possibilities remain to be addressed with future studies. However, from a technical viewpoint, the metabolic labeling method used in this study predominantly determines new protein synthesis. In contrast, measurements of the specific “FMRP targets” reflect the outcome of the expression level, which is bidirectionally determined by protein synthesis and protein stability/degradation. For stable proteins with a long half-life, suppression of new protein synthesis will not dramatically affect the expression level of the existing proteins.

FXS is a complex disorder. It is unlikely that a single treatment strategy will correct all aspects of symptoms. Regarding drug repurposing, several FDA-approved drugs, including minocycline (Siller and Broadie 2012), metformin (Dy et al., 2018), lovastatin (Osterweil et al., 2013), and trifluoperazine (Ding et al., 2020b), have shown certain therapeutic efficacy in the *Fmr1* KO mice. However, these drugs are not able to rescue all pathological outcomes. Repurposing new therapeutic reagents such as vorinostat will not only provide a new potential treatment choice but also expand the possibility of combination therapy. Regarding the transcriptome-based approach to identify new drug effects, other FDA-approved similarity drugs of trifluoperazine (Ding et al., 2020b) and vorinostat (Fig. 1; supplementary Tables 1–3) may be considered and examined in future studies.

In summary, we used unbiased transcriptome analysis to identify the new therapeutic potential of vorinostat as FXS treatment. We provide evidence to support the value of holistic transcriptome signature in drug repurposing. Vorinostat shows therapeutic effects on correcting cognitive and non-cognitive symptoms in FXS mice. This finding is directly relevant to clinical treatment potential and encourages future human trials.

## Supplementary Material

pyab081_suppl_Supplementary_Figure_S1

pyab081_suppl_Supplementary_Figure_S2

pyab081_suppl_Supplementary_Figure_S3

pyab081_suppl_Supplementary_Figure_S4

pyab081_suppl_Supplementary_Table_S1

pyab081_suppl_Supplementary_Table_S2

pyab081_suppl_Supplementary_Table_S3

pyab081_suppl_Supplementary_Table_S4

pyab081_suppl_Supplementary_Materials_S1

pyab081_suppl_Supplementary_Materials_S2
