# Supplementary figures and images for: Vorinostat Corrects Cognitive and Non-Cognitive Symptoms in a Mouse Model of Fragile X Syndrome
Source: Int J Neuropsychopharmacol. 2021 Nov 17;25(2):147–59. doi: 10.1093/ijnp/pyab081 (PMC8832232; doi:10.1093/ijnp/pyab081)

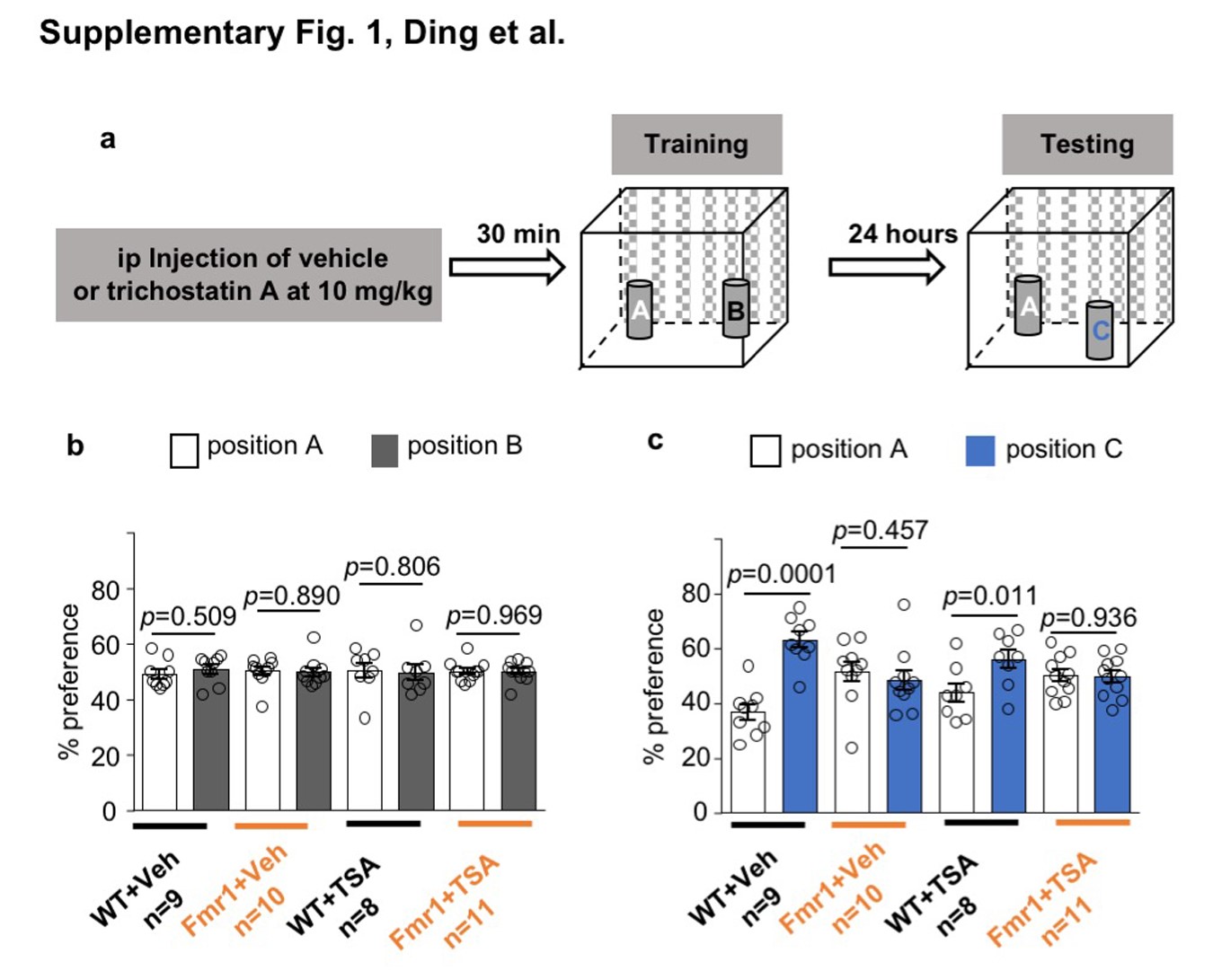

Supplement: pyab081_suppl_Supplementary_Figure_S1 [file pyab081_suppl_supplementary_figure_s1.jpeg]

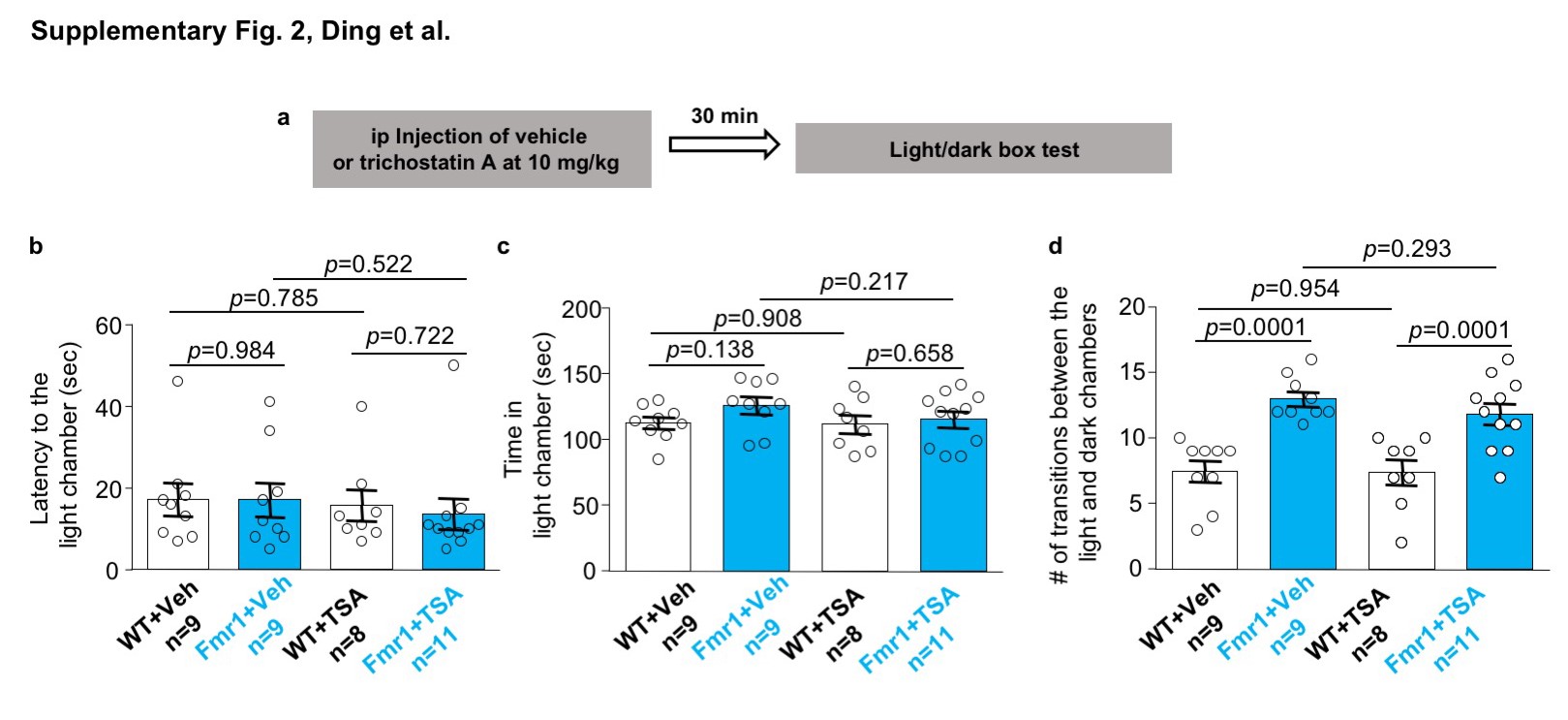

Supplement: pyab081_suppl_Supplementary_Figure_S2 [file pyab081_suppl_supplementary_figure_s2.jpeg]

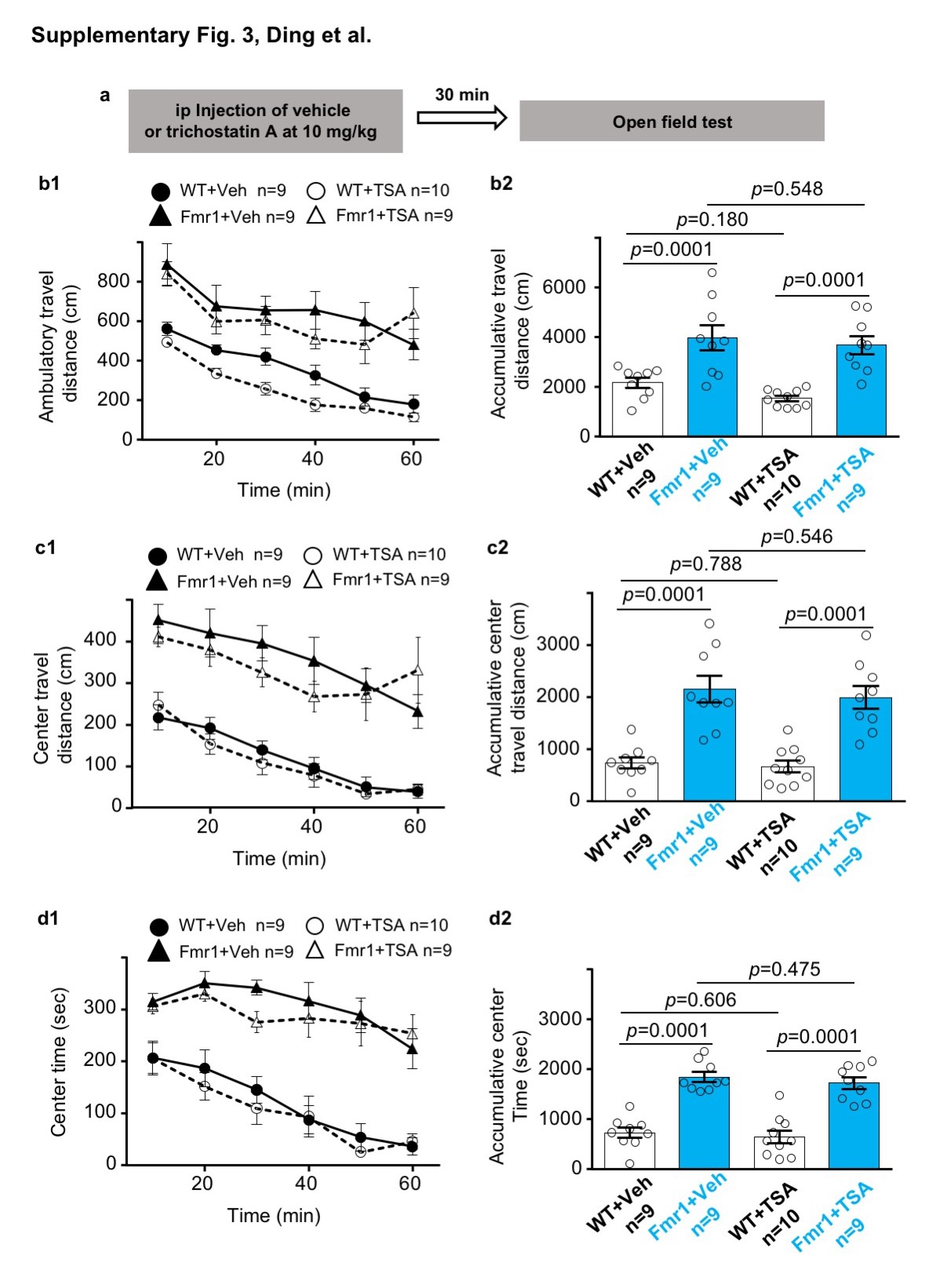

Supplement: pyab081_suppl_Supplementary_Figure_S3 [file pyab081_suppl_supplementary_figure_s3.jpeg]

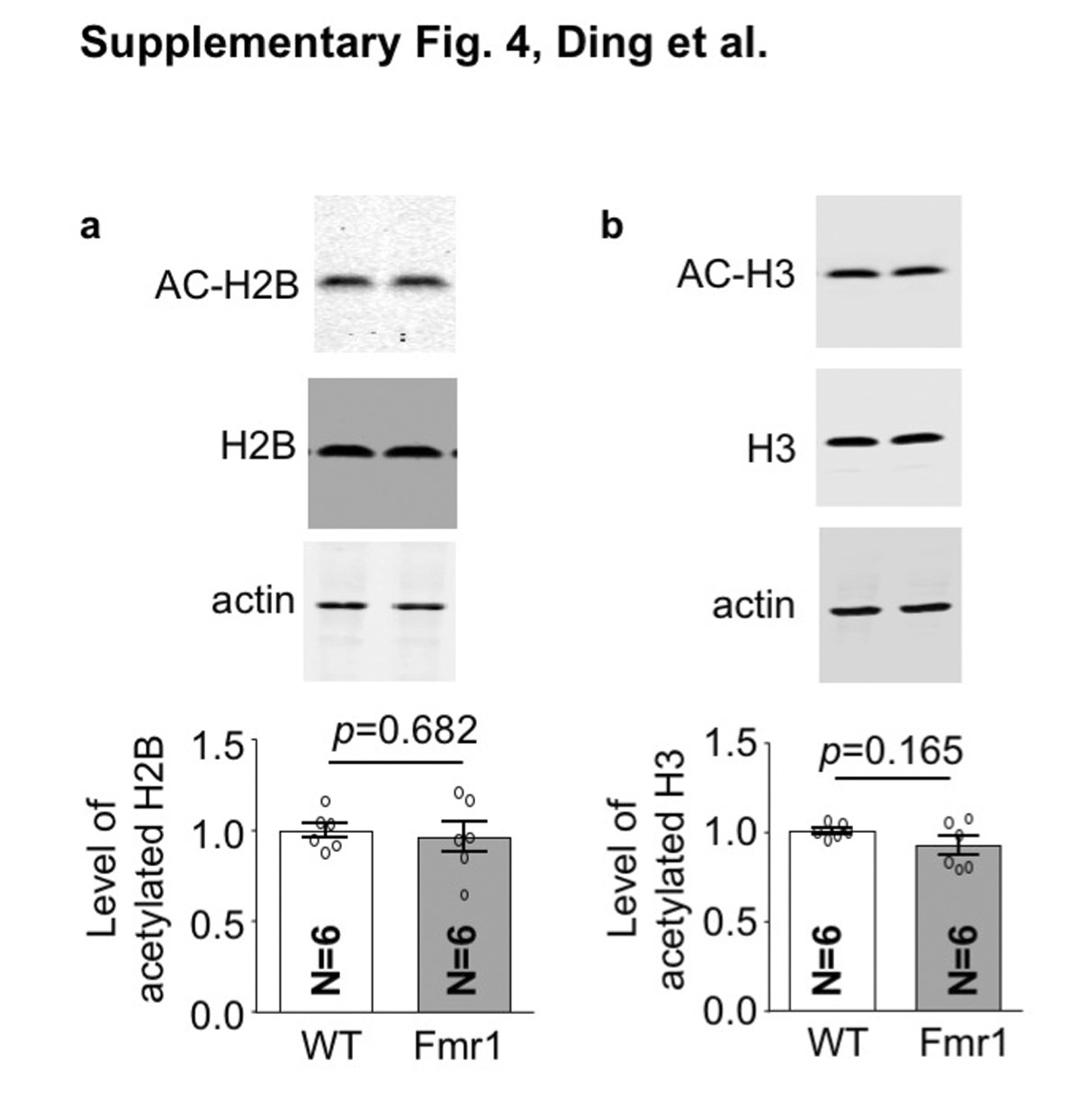

Supplement: pyab081_suppl_Supplementary_Figure_S4 [file pyab081_suppl_supplementary_figure_s4.jpeg]
