# Supplementary material for: Vorinostat Corrects Cognitive and Non-Cognitive Symptoms in a Mouse Model of Fragile X Syndrome
Source: Int J Neuropsychopharmacol. 2021 Nov 17;25(2):147–59. doi: 10.1093/ijnp/pyab081 (PMC8832232; doi:10.1093/ijnp/pyab081)
Supplement: pyab081_suppl_Supplementary_Table_S1 [file pyab081_suppl_supplementary_table_s1.pdf]

rank	cmap name	cell line	mean	n	enrichment	p-value	percent non-null
1	trichostatin A	MCF7	0.861	92	0.944	0	95
2	resveratrol	MCF7	0.364	6	0.937	0	100
3	15-delta prostaglandin J2	MCF7	0.298	8	0.805	0	100
4	trifluoperazine	MCF7	0.282	9	0.781	0	88
5	thioridazine	MCF7	0.259	11	0.781	0	90
6	tanespimycin	MCF7	0.239	36	0.707	0	86
7	fulvestrant	MCF7	0.228	21	0.683	0	85
8	LY-294002	MCF7	0.234	34	0.608	0	73
9	valproic acid	MCF7	0.233	31	0.475	0	54
10	moroxydine	MCF7	-0.826	2	-0.996	0.00004	100
11	lycorine	MCF7	-0.74	3	-0.978	0.00004	100
12	fluphenazine	MCF7	0.163	10	0.683	0.00004	70
13	troglitazone	MCF7	0.256	7	0.751	0.00018	85
14	phenoxybenzamine	MCF7	0.317	3	0.943	0.00022	100
15	pheneticillin	MCF7	-0.686	2	-0.989	0.00028	100
16	alvespimycin	MCF7	0.243	7	0.729	0.0003	85
17	racecadotril	MCF7	-0.693	2	-0.988	0.00036	100
18	prochlorperazine	MCF7	0.204	9	0.64	0.00044	66
19	geldanamycin	MCF7	0.198	10	0.599	0.00052	70
20	demecarium bromide	MCF7	-0.699	2	-0.983	0.00062	100
21	rottlerin	MCF7	0.278	3	0.922	0.00104	100
22	monastrol	MCF7	0.125	7	0.673	0.00104	85
23	rifabutin	MCF7	0.656	2	0.974	0.00107	100
24	disopyramide	MCF7	-0.682	2	-0.976	0.00125	100
25	iopromide	MCF7	-0.696	2	-0.975	0.00135	100
26	dimethadione	MCF7	-0.646	2	-0.971	0.00167	100
27	syrosingopine	MCF7	0.386	2	0.965	0.00211	100
28	aminophylline	MCF7	-0.585	2	-0.966	0.00254	100
29	ketorolac	MCF7	-0.578	2	-0.966	0.00254	100
30	ondansetron	MCF7	-0.628	2	-0.964	0.00292	100
31	estropipate	MCF7	-0.693	2	-0.963	0.00308	100
32	arachidonyltrifluoromethane	MCF7	-0.573	2	-0.959	0.00366	100
33	mycophenolic acid	MCF7	0.351	2	0.951	0.00439	100
34	rescinnamine	MCF7	0.326	2	0.95	0.00449	100
35	thiostrepton	MCF7	0.332	2	0.948	0.00497	100
36	mafenide	MCF7	-0.583	2	-0.95	0.00551	100
37	nortriptyline	MCF7	0.312	2	0.945	0.00567	100
38	depudecin	MCF7	0.344	2	0.945	0.00573	100
39	bufexamac	MCF7	0.35	2	0.943	0.00606	100
40	raloxifene	MCF7	0.277	3	0.85	0.00641	100
41	butyl hydroxybenzoate	MCF7	-0.347	3	-0.849	0.00681	66
42	methylbenzethonium chloride	MCF7	0.223	3	0.844	0.00749	100
43	sulconazole	MCF7	0.287	2	0.931	0.00891	100
44	hycanthone	MCF7	0.315	2	0.929	0.00972	100
45	homochlorcyclizine	MCF7	0.296	2	0.927	0.01022	100
46	nicergoline	MCF7	0.289	2	0.927	0.01022	100
47	5182598	MCF7	-0.5	2	-0.929	0.0105	50
48	lobeline	MCF7	-0.419	2	-0.927	0.01095	50
49	benzethonium chloride	MCF7	0.282	2	0.925	0.01097	100
50	mesoridazine	MCF7	-0.333	2	-0.927	0.01099	50
51	pimethixene	MCF7	0.279	2	0.923	0.01171	100

52	monorden	MCF7	0.156	12	0.44	0.01203	58
53	carcinine	MCF7	-0.39	2	-0.923	0.01205	50
54	clomifene	MCF7	0.295	2	0.922	0.01217	100
55	thioproperazine	MCF7	0.271	2	0.921	0.01223	100
56	quinostatin	MCF7	0.279	2	0.918	0.01314	100
57	5707885	MCF7	0.27	2	0.918	0.01336	100
58	fluspirilene	MCF7	0.267	2	0.917	0.01364	100
59	cefixime	MCF7	-0.445	2	-0.915	0.01453	50
60	protriptyline	MCF7	0.289	2	0.913	0.01511	100
61	harmol	MCF7	-0.319	2	-0.913	0.01529	50
62	ciclopirox	MCF7	0.266	2	0.912	0.01541	100
63	CP-645525-01	MCF7	0.267	2	0.912	0.01559	100
64	cyclopenthiazide	MCF7	-0.347	2	-0.911	0.01589	50
65	desipramine	MCF7	0.289	2	0.91	0.0163	100
66	clomipramine	MCF7	0.265	2	0.91	0.01648	100
67	mefloquine	MCF7	0.259	2	0.91	0.01664	100
68	suprofen	MCF7	-0.298	2	-0.909	0.0167	50
69	zidovudine	MCF7	-0.326	2	-0.908	0.01686	50
70	apramycin	MCF7	-0.33	2	-0.906	0.01787	50
71	prenylamine	MCF7	0.25	2	0.906	0.01813	100
72	amoxapine	MCF7	0.282	2	0.905	0.01837	100
73	withaferin A	MCF7	0.257	2	0.905	0.01853	100
74	epivincamine	MCF7	-0.395	2	-0.903	0.01865	50
75	perhexiline	MCF7	0.259	2	0.903	0.0194	100
76	(+/-)-catechin	MCF7	-0.281	2	-0.901	0.01966	50
77	nitrofurantoin	MCF7	0.25	2	0.901	0.02026	100
78	cinoxacin	MCF7	-0.328	2	-0.898	0.02072	50
79	loperamide	MCF7	0.179	3	0.783	0.02097	66
80	parthenolide	MCF7	0.253	2	0.898	0.02167	100
81	dosulepin	MCF7	0.247	2	0.897	0.02195	100
82	colforsin	MCF7	0.271	2	0.895	0.02272	100
83	talampicillin	MCF7	-0.336	2	-0.894	0.02276	50
84	nordihydroguaiaretic acid	MCF7	0.164	8	0.499	0.02319	62
85	0317956-0000	MCF7	0.103	4	0.674	0.02485	50
86	corticosterone	MCF7	0.263	2	0.889	0.02559	100
87	terfenadine	MCF7	0.255	2	0.887	0.0263	100
88	etoposide	MCF7	0.29	2	0.886	0.02702	100
89	5224221	MCF7	0.252	2	0.885	0.0273	100
90	tonzonium bromide	MCF7	0.229	2	0.883	0.02811	100
91	pentoxyverine	MCF7	-0.236	2	-0.881	0.02831	50
92	meclocycline	MCF7	-0.452	2	-0.879	0.02931	50
93	metanephrine	MCF7	0.22	2	0.879	0.02936	100
94	esculin	MCF7	-0.246	2	-0.87	0.03356	50
95	pizotifen	MCF7	0.217	2	0.869	0.03479	100
96	bepridil	MCF7	0.214	2	0.867	0.03608	100
97	alimemazine	MCF7	0.219	2	0.867	0.0364	100
98	NS-398	MCF7	0.231	2	0.866	0.03672	100
99	butoconazole	MCF7	0.228	2	0.865	0.0369	100
100	cefuroxime	MCF7	-0.38	2	-0.864	0.03696	50
101	pyrvinium	MCF7	0.183	4	0.647	0.03724	75
102	digoxigenin	MCF7	0.122	3	0.733	0.03754	66
103	clenbuterol	MCF7	-0.36	3	-0.736	0.03768	66

104	CP-690334-01	MCF7	0.258	4	0.644	0.03867	75
105	probucol	MCF7	-0.277	4	-0.637	0.04201	50
106	disulfiram	MCF7	0.224	2	0.855	0.04284	100
107	cinchocaine	MCF7	0.194	2	0.846	0.0476	100
108	sulfathiazole	MCF7	-0.374	2	-0.842	0.04996	50
