# Supplementary material for: Vorinostat Corrects Cognitive and Non-Cognitive Symptoms in a Mouse Model of Fragile X Syndrome
Source: Int J Neuropsychopharmacol. 2021 Nov 17;25(2):147–59. doi: 10.1093/ijnp/pyab081 (PMC8832232; doi:10.1093/ijnp/pyab081)
Supplement: pyab081_suppl_Supplementary_Table_S2 [file pyab081_suppl_supplementary_table_s2.pdf]

rank	cmap name	cell line	mean	n	enrichment	p-value	percent non-null
1	trichostatin A	PC3	0.81	55	0.976	0	98
2	thioridazine	PC3	0.289	5	0.925	0.00002	100
3	LY-294002	PC3	0.181	12	0.665	0.00002	75
4	Prestwick-675	PC3	-0.909	2	-0.998	0.00004	100
5	scriptaid	PC3	0.772	2	0.992	0.00006	100
6	AH-6809	PC3	-0.756	2	-0.995	0.00008	100
7	trifluoperazine	PC3	0.307	3	0.96	0.0001	100
8	lidocaine	PC3	-0.681	2	-0.99	0.00026	100
9	fluphenazine	PC3	0.27	3	0.942	0.00026	100
10	valproic acid	PC3	0.257	10	0.595	0.0006	70
11	calcium folinate	PC3	-0.612	2	-0.978	0.00101	100
12	MS-275	PC3	0.545	2	0.975	0.00101	100
13	15-delta prostaglandin J2	PC3	0.271	3	0.92	0.00112	100
14	MG-262	PC3	0.343	2	0.968	0.00173	100
15	josamycin	PC3	-0.574	2	-0.971	0.00175	100
16	astemizole	PC3	0.333	2	0.965	0.00207	100
17	beta-escin	PC3	0.319	2	0.964	0.00221	100
18	mefloquine	PC3	0.296	2	0.962	0.00256	100
19	dilazep	PC3	0.332	2	0.96	0.00266	100
20	helveticoside	PC3	0.307	2	0.959	0.00294	100
21	withaferin A	PC3	0.278	2	0.957	0.0033	100
22	azacyclonol	PC3	0.271	2	0.955	0.00366	100
23	methylbenzethonium chloride	PC3	0.284	2	0.953	0.00404	100
24	resveratrol	PC3	0.271	2	0.952	0.00416	100
25	perphenazine	PC3	0.262	2	0.948	0.00497	100
26	prochlorperazine	PC3	0.254	3	0.859	0.00531	100
27	mianserin	PC3	0.249	2	0.942	0.00638	100
28	disulfiram	PC3	0.262	2	0.941	0.00672	100
29	cloperastine	PC3	0.232	2	0.935	0.00817	100
30	alexidine	PC3	0.248	2	0.934	0.00819	100
31	lomustine	PC3	0.233	2	0.929	0.00962	100
32	loperamide	PC3	0.219	2	0.92	0.01272	100
33	captopril	PC3	-0.337	2	-0.916	0.01433	50
34	CP-690334-01	PC3	0.2	4	0.708	0.01494	50
35	PHA-00846566E	PC3	-0.415	2	-0.912	0.01553	50
36	famotidine	PC3	-0.322	2	-0.91	0.01638	50
37	spironolactone	PC3	0.211	2	0.91	0.01642	100
38	nomifensine	PC3	-0.334	2	-0.909	0.01674	50
39	bromopride	PC3	-0.315	2	-0.904	0.01855	50
40	chlorcyclizine	PC3	0.209	2	0.903	0.0194	100
41	5194442	PC3	0.201	2	0.899	0.0207	100
42	AR-A014418	PC3	-0.397	2	-0.898	0.02072	50
43	riluzole	PC3	-0.389	2	-0.896	0.02157	50
44	hydralazine	PC3	-0.294	2	-0.894	0.02235	50
45	SC-19220	PC3	-0.436	2	-0.893	0.02304	50
46	tolfenamic acid	PC3	-0.327	2	-0.882	0.02803	50
47	PHA-00816795	PC3	-0.45	2	-0.882	0.02819	50
48	mebendazole	PC3	0.206	2	0.882	0.02831	100
49	0198306-0000	PC3	-0.402	2	-0.879	0.02925	50
50	antimycin A	PC3	0.217	2	0.877	0.03026	100
51	CP-863187	PC3	-0.396	2	-0.875	0.03109	50

52	hydrastinine	PC3	-0.321	2	-0.871	0.03278	50
53	PNU-0293363	PC3	-0.303	2	-0.866	0.03626	50
54	16,16-dimethylprostaglandin E2	PC3	0.188	2	0.866	0.03648	100
55	troglitazone	PC3	0.153	4	0.647	0.03712	75
56	acenocoumarol	PC3	-0.372	2	-0.86	0.03899	50
57	orphenadrine	PC3	0.215	2	0.861	0.03901	100
58	ethosuximide	PC3	-0.372	2	-0.855	0.04191	50
59	oxetacaine	PC3	0.188	2	0.856	0.04231	100
60	gallamine triethiodide	PC3	-0.293	2	-0.854	0.04241	50
61	tropicamide	PC3	-0.315	2	-0.852	0.0437	50
62	triamcinolone	PC3	-0.35	2	-0.845	0.04825	50
