# Supplementary material for: Vorinostat Corrects Cognitive and Non-Cognitive Symptoms in a Mouse Model of Fragile X Syndrome
Source: Int J Neuropsychopharmacol. 2021 Nov 17;25(2):147–59. doi: 10.1093/ijnp/pyab081 (PMC8832232; doi:10.1093/ijnp/pyab081)
Supplement: pyab081_suppl_Supplementary_Table_S3 [file pyab081_suppl_supplementary_table_s3.pdf]

rank	cmap name	cell line	mean	n	enrichment	p-value	percent non-null
1	trichostatin A	HL60	0.573	34	0.965	0	100
2	valproic acid	HL60	0.423	14	0.81	0	85
3	LY-294002	HL60	0.291	13	0.793	0	84
4	tanespimycin	HL60	0.187	12	0.759	0	83
5	15-delta prostaglandin J2	HL60	0.351	3	0.964	0.00006	100
6	genistein	HL60	0.297	3	0.96	0.0001	100
7	geldanamycin	HL60	0.242	3	0.942	0.00024	100
8	tretinoin	HL60	0.184	5	0.806	0.00062	80
9	fluphenazine	HL60	0.102	4	0.749	0.0076	50
10	trifluoperazine	HL60	0.19	4	0.742	0.00855	75
11	sirolimus	HL60	0.102	10	0.494	0.00907	50
12	prochlorperazine	HL60	0.192	4	0.724	0.0116	75
13	thioridazine	HL60	0.184	4	0.695	0.0181	75
14	troglitazone	HL60	0.163	4	0.678	0.02335	75
15	raloxifene	HL60	0.158	2	0.888	0.02622	100
16	sodium phenylbutyrate	HL60	0.197	2	0.886	0.02694	100
