# Supplementary material for: Vorinostat Corrects Cognitive and Non-Cognitive Symptoms in a Mouse Model of Fragile X Syndrome
Source: Int J Neuropsychopharmacol. 2021 Nov 17;25(2):147–59. doi: 10.1093/ijnp/pyab081 (PMC8832232; doi:10.1093/ijnp/pyab081)
Supplement: pyab081_suppl_Supplementary_Table_S4 [file pyab081_suppl_supplementary_table_s4.pdf]

Supplementary Table 4

**Experiment**	**Data presentation**	**Number of litters involved**
object location memory	Fig. 2	12
object location memory	Fig. 6	10
object location memory	Supplementary Fig. 1	11
passive avoidance	Fig. 2d	7
passive avoidance	Fig. 2e	8
light/dark test	Fig. 3	13
light/dark test	Fig. 6	13
light/dark test	Supplementary Fig. 2	13
open field test	Fig. 3	10
open field test	Supplementary Fig. 3.	12
social interaction test	Fig. 4a	12
social interaction test	Fig. 4b	8
protein synthesis analysis	Fig. 5	2
in vivo histone acetylation	Fig. 7	12
in vitro acetylation	Supplementary Fig. 4	2
