# Supplementary material for: Vorinostat Corrects Cognitive and Non-Cognitive Symptoms in a Mouse Model of Fragile X Syndrome
Source: Int J Neuropsychopharmacol. 2021 Nov 17;25(2):147–59. doi: 10.1093/ijnp/pyab081 (PMC8832232; doi:10.1093/ijnp/pyab081)
Supplement: pyab081_suppl_Supplementary_Materials_S1 [file pyab081_suppl_supplementary_materials_s1.docx]

**SUPPLEMENTARY FIGURE LEGENDS**

**Supplementary Fig 1** High dose trichostatin A does not improve object location memory in *Fmr1* KO mice. **a.** Wild type (WT) and *Fmr1* KO (Fmr1) mice were injected with vehicle (Veh) or trichostatin A at 10 mg/kg (TSA), and examined for object location memory. **b.** Mouse preference to the object at locations A and B during training. **c.** Mouse preference to the object at locations A and C during testing. Data are presented as mean +/- SEM. The *p*-values were determined by three-way ANOVA followed by *post hoc* pairwise comparison with Holm-Sidak adjustment.

**Supplementary Fig 2** High dose trichostatin A does not attenuate the repetitive transition behavior in *Fmr1* KO mice. **a.** Wild type (WT) and *Fmr1* KO (Fmr1) mice were injected with vehicle (Veh) or trichostatin A at 10 mg/kg (TSA). 30 min later, mice were subjected to a light/dark box test. During the light/dark box test, the mice were allowed to make transitional moves between the light and the dark chamber. The latency to exit the dark chamber (**b**), time spent in the light chamber (**c**), and number of transitional moves between the light and dark chambers (**d**) were recorded. Data are presented as mean +/- SEM. The *p*-values were determined by two-way ANOVA followed by *post hoc* pairwise comparison with Holm-Sidak adjustment.

**Supplementary Fig 3** High dose trichostatin A does not affect behavior in the open field in the *Fmr1* KO mice. **a.** Wild type (WT) and *Fmr1* KO (Fmr1) mice were injected with vehicle (Veh) or trichostatin A at 10 mg/kg (TSA). 30 min later, mice were subjected to the open field test. During the 60-min open field test, ambulatory travel distance within the whole arena (**b**), ambulatory travel distance in the center area (**c**), and time spent in the center area (**d**) were recorded. Activities for each of the 10 min bin (**b1, c1** and **d1**) and accumulative activity during the whole 60 min testing (**b2, c2** and **d2**) are presented as mean +/- SEM. The *p*-values were determined by two-way ANOVA followed by *post hoc* pairwise comparison with Holm-Sidak adjustment.

**Supplementary Fig 4** *Fmr1* deficiency does not affect histone acetylation in cultured neurons. Samples collected from DIV 14 WT and *Fmr1* KO hippocampal neurons (n=6; triplicates from 2 independent primary neuronal cultures) were analyzed for the level of histone acetylation. Western blot was used to determine the level of acetylated H2B (**a**) and acetylated H3 (**b**), which were normalized to the total level of the respective histone proteins. Data are presented as mean +/- SEM. Data were analyzed by Student’s t-test.
