# Supplementary material for: Vorinostat Corrects Cognitive and Non-Cognitive Symptoms in a Mouse Model of Fragile X Syndrome
Source: Int J Neuropsychopharmacol. 2021 Nov 17;25(2):147–59. doi: 10.1093/ijnp/pyab081 (PMC8832232; doi:10.1093/ijnp/pyab081)
Supplement: pyab081_suppl_Supplementary_Materials_S2 [file pyab081_suppl_supplementary_materials_s2.docx]

**SUPPLEMENTARY TABLE LEGENDS**

**Supplementary Table 1** vorinostat similarity compounds screened by DEGs induced by vorinostat in MCF7 cells. The *p*-value determines the ranking of the similarity compounds/drugs; compounds/drugs are listed with their *p*-values in ascending order.

**Supplementary Table 2** vorinostat similarity compounds screened by DEGs induced by vorinostat in PC3 cells. The *p*-value determines the ranking of the similarity compounds/drugs; compounds/drugs are listed with their *p*-values in ascending order.

**Supplementary Table 3** vorinostat similarity compounds screened by DEGs induced by vorinostat in HL60 cells. The *p*-value determines the ranking of the similarity compounds/drugs; compounds/drugs are listed with their *p*-values in ascending order.

**Supplementary Table 4** number of litters of mice used for data collection. All experiments involve mice from multiple litters to avoid pseudo repeat. The information of litters used for each specific experiment and data presentation is listed.
